# Application of a Persistent Heparin Treatment Inhibits the Malignant Potential of Oral Squamous Carcinoma Cells Induced by Tumor Cell-Derived Exosomes

**DOI:** 10.1371/journal.pone.0148454

**Published:** 2016-02-05

**Authors:** Shinya Sento, Eri Sasabe, Tetsuya Yamamoto

**Affiliations:** Department of Oral and Maxillofacial Surgery, Kochi Medical School, Kochi University, Kohasu, Oko-cho, Nankoku-city, Kochi 783–8505, Japan; China Medical University, TAIWAN

## Abstract

Exosomes are 30–100 nm-sized membranous vesicles, secreted from a variety of cell types into their surrounding extracellular space. Various exosome components including lipids, proteins, and nucleic acids are transferred to recipient cells and affect their function and activity. Numerous studies have showed that tumor cell-derived exosomes play important roles in tumor growth and progression. However, the effect of exosomes released from oral squamous cell carcinoma (OSCC) into the tumor microenvironment remains unclear. In the present study, we isolated exosomes from OSCC cells and investigated the influence of OSCC cell-derived exosomes on the tumor cell behavior associated with tumor development. We demonstrated that OSCC cell-derived exosomes were taken up by OSCC cells themselves and significantly promoted proliferation, migration, and invasion through the activation of the PI3K/Akt, MAPK/ERK, and JNK-1/2 pathways *in vitro*. These effects of OSCC cell-derived exosomes were obviously attenuated by treatment with PI3K, ERK-1/2, and JNK-1/2 pharmacological inhibitors. Furthermore, the growth rate of tumor xenografts implanted into nude mice was promoted by treatment with OSCC cell-derived exosomes. The uptake of exosomes by OSCC cells and subsequent tumor progression was abrogated in the presence of heparin. Taken together, these data suggest that OSCC cell-derived exosomes might be a novel therapeutic target and the use of heparin to inhibit the uptake of OSCC-derived exosomes by OSCC cells may be useful for treatment.

## Introduction

Oral cancer accounts for 2%–3% of all human malignancies and is trending upward yearly [1]. Regardless of recent advancements in many therapeutic strategies, oral cancer remains associated with recurrence and deterioration. Oral squamous cell carcinoma (OSCC) accounts for approximately 90% of cancer types. Thus, the performance of treatments for oral cancer needs to be improved.

Exosomes, which are small membrane vesicles that originate from multi-vesicular bodies, 30–100 nm in diameter, are released by a variety of mammalian cells into the extracellular space and are taken up by recipient cells [2, 3]. Exosomes contain lipids, proteins, and nucleic acids from their cell of origin, which are transferred to recipient cells and affect their function and activity [4]. It has been reported that exosomes are loaded into recipient cells by mechanisms such as cell fusion, receptor-mediated uptake, and internalization [5]. Thus, exosomes may be involved in a number of physiological and pathological processes, including cancer through the regulation of cell-cell communication [6–9]. Numerous tumor cells produce exosomes, which are emerging as potential tools for the early detection or control of human cancers, including head and neck cancer [10–12]. Regarding the role of tumor cell-derived exosomes, some studies have demonstrated that tumor cell-derived exosomes possess anti-tumor properties by inducing apoptosis of tumor cells or by enhancing anti-tumor immunity [13–15]. However, tumor cell-derived exosomes may promote tumor progression by exhibiting immunosuppressive properties, facilitating tumor invasion and metastasis, stimulating tumor cell proliferation, or inducing drug resistance [8, 16–20]. These contradictory findings lead us to examine the exact function of OSCC cell-derived exosomes in cancer development.

Targeting tumor cell-secreted exosomes has therapeutic potential. For example, during the process of exosomes production and secretion, blockage of Rab27 and sphingomyelinases has a suppressive role in tumor formation, angiogenesis, or metastasis [21–23]. Effects of targeting elements of the exosome cargo, such as micro RNA (miRNA) or kinases, were also reported [24]. Furthermore, blockage of the exosome-uptake pathways may be effective for cancer therapy. Heparan sulfate proteoglycans (HSPGs) function as receptors of cancer cell-derived exosomes. Christianson *et al*. indicated that the HSPG-dependent uptake route was highly relevant for the biological activity of exosomes and heparin treatment effectively inhibited exosome-mediated tumor development [25]. Therefore, we focused on heparin as a clinically applicable drug and explored whether heparin could inhibit the tumor effects induced by OSCC cell-derived exosomes.

We found that OSCC cell-derived exosomes promoted tumor growth and progression. Moreover, OSCC cell treatment with heparin inhibited exosome uptake by recipient cells as well as tumor growth and progression induced by OSCC cell-derived exosomes *in vitro* and *in vivo*. Thus, the results of the present study provide novel therapeutic strategies, targeting OSCC cell-derived exosomes critical for OSCC growth and progression.

## Materials and Methods

### Cell culture

OSCC cell lines (OSC-3 and -4 cells) were established in our laboratory from patients with OSCC and have been described previously [26]. OSCC cells were cultured in Dulbecco’s modified Eagle’s medium (DMEM; Nissui Pharmaceutical Co. Ltd., Tokyo, Japan) supplemented with 10% (v/v) fetal bovine serum (FBS), 10 mM glutamine, 100 units/mL of penicillin, and 100 μg/mL of streptomycin (Invitrogen, Carlsbad, CA, USA) at 37°C in a humidified 5% CO_2_/95% air atmosphere.

### Exosome isolation

OSCC cells (2 × 10^6^ cells/10 cm dish) were cultured in conventional culture medium for 24 h. The medium was then replaced with an exosome-depleted FBS-containing (EXO-FBS, System Biosciences, Mountain View, CA, USA) medium and cultured for 48 h. OSCC cell-derived exosomes were isolated using total exosome isolation kit (Invitrogen) according to the manufacturer’s protocol. Briefly, cell culture supernatants were harvested and centrifuged at 2,000 × *g* for 30 min to remove cells and cell debris. Next, we added the reagent to the supernatants and the mixture was refrigerated overnight. The mixture was then centrifuged at 10,000 × *g* for 60 min and the supernatants were removed. The exosome pellet was re-suspended in phosphate buffered saline (PBS) and the protein concentration was determined using a BCA protein assay kit (Pierce Biotechnology, Rockford, IL, USA). LY294002, PD98059, and SP600125 were supplied by Calbiochem (La Jolla, CA, USA). Heparin was obtained from Nacalai Tesque (Kyoto, Japan). Treatment details are provided in the Figure Legends.

### Transmission electron microscopy

Purified exosomes were fixed with paraformaldehyde to copper mesh Formvar grids (ProSciTech, Townsville, QLD, Australia) and immunolabeled with a mouse monoclonal anti-human CD9 antibody (BD Biosciences, San Jose, CA, USA) and a gold-labeled (10 nm) goat anti-mouse IgG secondary antibody (Sigma-Aldrich, St. Louis, MO, USA). Grids were incubated in 1% glutaraldehyde in PBS (pH 7.4) and negatively stained by 0.5% uranyl acetate. Samples were observed using the JEOL JEM-1400 Plus Transmission Electron Microscope (JEOL, Japan)

### Exosome labeling and cellular uptake

Purified exosomes were labeled with PKH26 (Sigma-Aldrich), according to the manufacturer’s protocol with minor modifications. Briefly, 1 μL of PKH26 was added to 100 μg of OSCC-derived exosome pellets in a total volume of 400 μL Diluent C and incubated for 5 min at room temperature. The labeling reaction was stopped by adding an equal volume of 1% BSA. Labeled exosomes were ultra-centrifuged at 10,000 × *g* for 60 min at 4°C. The supernatant was then removed and the pellet was re-suspended in 20 μL PBS. OSCC cells (1 × 10^4^ cells/well) were cultured in Nunc Lab Tek 8-well chamber slides (Thermo Fisher Scientific, Waltham, MA, USA) for 24 h and pretreated with or without 10 μg/mL heparin for 1 h. Cells were then incubated with 100 μg PKH26-labeled exosomes in the presence or absence of 10 μg/mL heparin for 1, 4, 8, and 16 h at 37°C with 5% CO_2_. After incubation, cells were washed twice with PBS and fixed with 200 μL Fixing Solution (Cell Biolabs, San Diego, CA, USA) for 10 min at room temperature. The cells were washed twice with PBS, 200 μL of DAPI solution were added (Cell Biolabs), and the cells were incubated for 15 min at room temperature. Cellular uptake of OSCC-derived exosomes was observed under a confocal laser microscope.

### Cell proliferation assay (MTT assay and CyQUANT cell proliferation assay)

Cell proliferation was estimated by the 3-(4,5-dimethylthiazol-2-yl)-2,5-diphenyl-2H-tetrazolium bromide (MTT) colorimetric assay and CyQUANT Cell Proliferation Assay (invitrogen). About MTT assay, cells (3 × 10^3^ cells/well) were cultured in a 96-well microplate in the presence or absence of OSCC-derived exosomes. After each treatment, the cells were washed with 200 μL of PBS and incubated with 5 mg/mL MTT solution (Sigma-Aldrich) at 37°C for 4 h. The supernatants were then removed and the formazan crystals in each well were solubilized by the addition of 200 μL of dimethyl sulfoxide for 30 min. The colored formazan product was measured using a plate reader at a wavelength of 570 nm. About CyQUANT cell proliferation assay, cells (3 × 10^3^ cells/well) were cultured in a 96-well microplate in the presence or absence of OSCC-derived exosomes. The 2× detection reagent was prepared according to the manufacturer's protocol. After each treatment, 100 μL of CyQUANT Cell Proliferation Assay reagent was added to each well. After incubation for 30 minutes at 37°C, fluorescence was measured (excitation 485 nm, emission 538 nm) using a plate reader. Experiments were repeated three times in triplicate for each experiment.

### Wound healing assay

Wound healing assays were performed using CytoSelect^™^ 24-Well Wound Healing Assay (Cell Biolabs). Briefly, OSCC cells were seeded in a 24-well plate containing proprietary treated plastic inserts at 2.5 × 10^4^ cells/well and cultured for 24 h. The inserts were then removed and cells were cultured with serum-free DMEM in the presence or absence of OSCC cells-derived exosomes for 10 h. After staining the cells with Cell Stain Solution for 15 min, we measured the percentage of closure of the wound field by light microscopy. Experiments were repeated three times in triplicate for each experiment.

### Invasion assay

The cell invasive potential was examined using a BioCoat Matrigel Invasion Chamber kit (BD Biosciences) and the invasive activity was determined according to the manufacturer's instructions. Briefly, 7.5 × 10^4^ cells were added to the transwell insert chamber with a filter coated with Matrigel. In the lower compartment, 750 μL DMEM containing 10% (v/v) FBS was used as the chemoattractant. The cells were incubated with or without OSCC-derived exosomes for 18 h at 37°C under 5% CO_2_/95% air atmosphere. The inserts were removed and non-invading cancer cells remaining on the upper side of the filter were scraped off. Cells that invaded into the lower side of the filter were then stained with Diff-Quick and microscopically observed and counted in five fields at 200× magnification. The invasive activity of cancer cells was expressed as the mean number of cells that invaded to the lower side of the filter and the results are presented as mean ± SD of cells per field. Experiments were repeated three times in triplicate for each experiment.

### Proteome profiler array

To identify exosome-activated signal transduction molecules, we used the Proteome Profiler^™^ Human Phospho-Kinase Array Kit (R&D Systems, Minneapolis, MN, USA) according to the manufacturer's instructions. OSCC cells were seeded in a 10 cm dish at 8 × 10^5^ cells and cultured in the presence or absence of 100 μg exosomes for 24 h. The cells were pelleted and lysed in lysis buffer. The densitometric analysis of the arrays was performed using the TotalLab TL100 software (Nonlinear Dynamics Ltd, Newcastle upon Tyne, UK).

### Protein extraction and western blot analysis

OSCC cells or OSCC cell-derived exosomes were lysed in RIPA buffer [27]. Protein concentrations were determined using a BCA protein assay kit. Extracted proteins (50 μg/lane) were separated by SDS-polyacrylamide gel electrophoresis and transferred onto an Immobilon-P membrane (Immobilon, Millipore Corporation, Bedford, MA, USA). Blocking was performed in Tris-buffered saline containing 5% (w/v) skim milk powder and 0.1% (v/v) Tween-20. The membranes were probed with antibodies (Abs) against CD9, CD63, Cytochrome C, Annexin II (BD Biosciences), Calnexin (EMD Millipore, Temecula, CA, USA), Rab5B, total Akt, total p44/p42 MAPKs, total JNK, β-actin (Santa Cruz Biotechnology, Santa Cruz, CA, USA), phosphorylated (Ser473) Akt (pAkt), phosphorylated (Thr202/Tyr204) p44/p42 MAPK (pERK), and phosphorylated JNK (pJNK). The detection was performed using an ECL system (Amersham, Piscataway, NJ, USA).

### Xenograft tumor model

OSC-4 cells (2 × 10^5^/0.05 mL) with or without OSC-4-derived exosomes were subcutaneously injected into the backs of 5-week-old BALB/c nude mice (Japan Clea, Osaka, Japan). To examine the effects of heparin, mice were surgically implanted subcutaneously in the intrascapular area with mini-osmotic pumps (Alzet, Cupertino, CA, USA) filled under sterile conditions, in accordance with the manufacturer's instructions, with either 200 μL of 10 μg/mL heparin or isotonic saline. Pumps had a mean flow rate of 0.25 μL/h. Tumor size was measured with calipers and the volume was calculated as follows: (length × width^2^) × 0.5 every 3 days [28, 29]. Mice were euthanized on day 27. All experimental procedures performed on mice were approved by the Institutional Animal Care and Use Committee of Kochi Medical School. (Permission number of this experiment: I-00021).

### Statistical analysis

Results are expressed as the mean ± SD. Differences were compared using Mann-Whitney's U-test, and *p*-values less than 0.05 were considered statistically significant.

## Results

### Isolation and characterization of OSCC cell-derived exosomes

**Fig 1:**
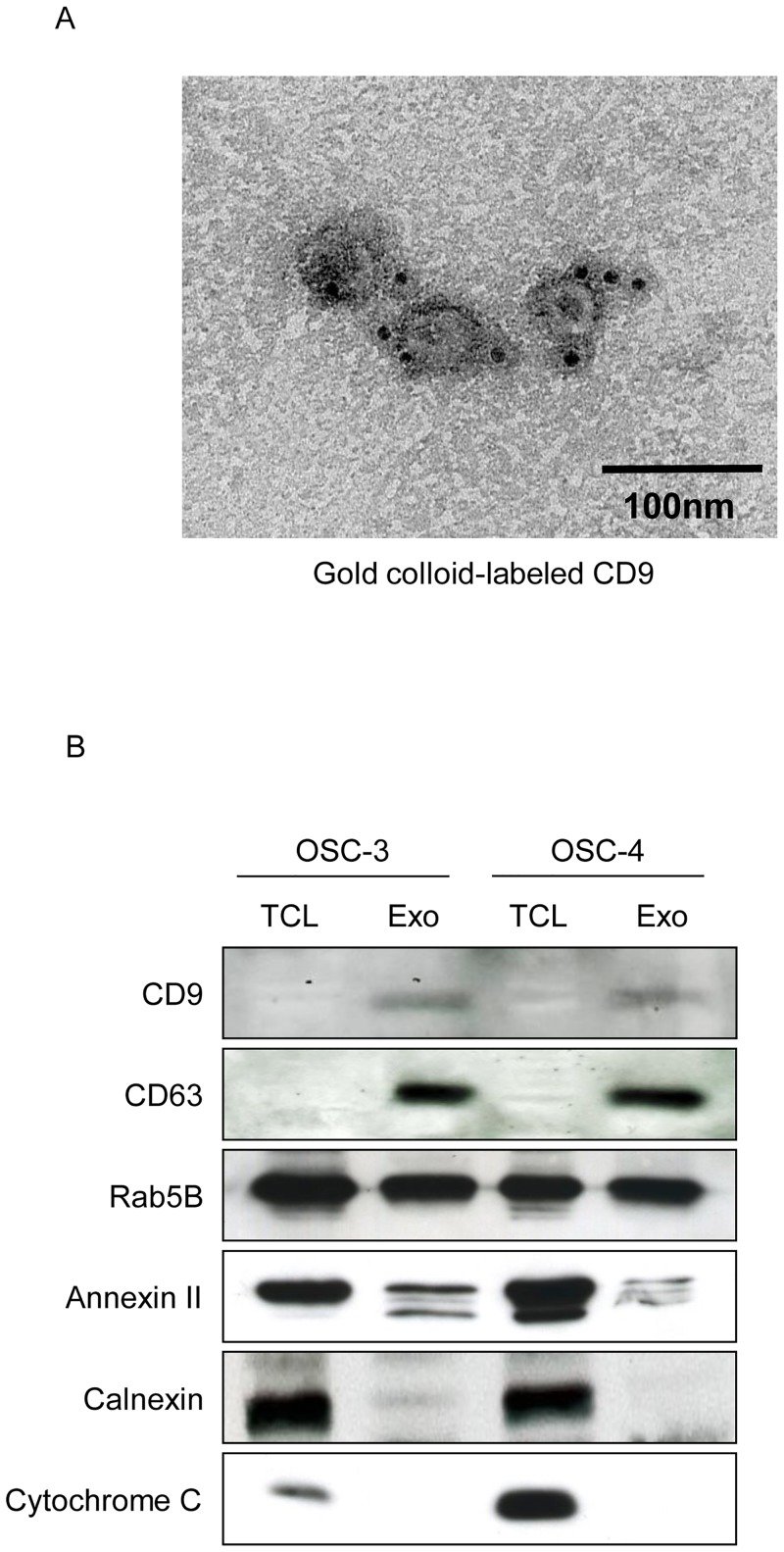
Characterization of OSCC cell-derived exosomes. (A) Immunolabeling of exosomes derived from OSC-4 cells. Purified exosomes were transferred to copper grids and immunostained with mouse anti-human CD63 followed by rabbit anti-mouse antibody and colloidal gold coated with protein A (10 nm). Samples were stained with uranyl acetate and analyzed by transmission electron microscopy. Scale bar = 100 nm. (B) Total cell lysate (TCL) and exosomes (Exo) from OSC-3 and OSC-4 cells were analyzed by western blotting with antibodies against CD9, CD63, Rab5B, Annexin II, Calnexin, and Cytochrome C.

To characterize OSCC cell-derived exosomes, we used transmission electron microscopy. Purified exosomes from OSC-3 and OSC-4 cells presented a round-shaped vesicular membrane structure. They were 30–100 nm in a diameter and positively immunolabeled with CD9-specific gold particle-conjugated antibodies (Fig 1A). In addition, western blot analysis demonstrated that tetraspanins CD9 and CD63, Rab5B, and Annexin II which are used markers for exosomes, were expressed on OSC-3 and OSC-4 cell-derived exosomes. On the other hands, we did not detect the expression of Calnexin or Cytochrome C in the exosome preparation (Fig 1B).

### OSCC cell-derived exosomes are taken up by the host cells

**Fig 2:**
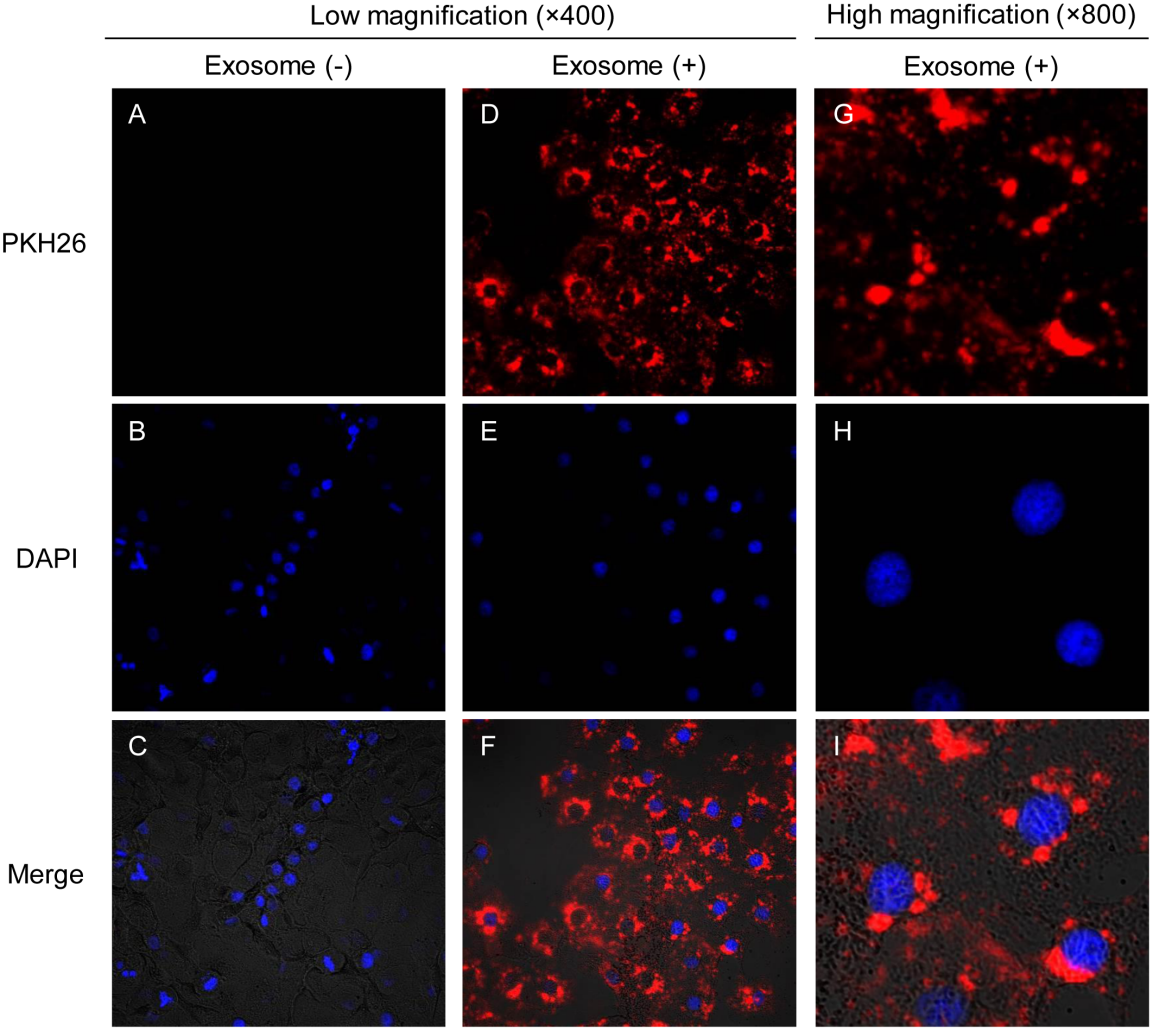
Cellular internalization of OSCC cell-derived exosomes into OSCC cells. OSC-4 cells were incubated in the presence or absence of 100 μg of PKH26 (red)-labeled exosomes from OSC-4 cells for 4 h and analyzed by confocal microscopy. Nuclei were stained with DAPI (blue). (A-C) Low magnification images of OSC-4 cells without exosomes (400 ×). (D-F) Low magnification images of OSC-4 cells with exosomes (400 ×). (G-I) High magnification images of OSC-4 cells incubated with exosomes (800 ×).

To study the uptake of isolated exosomes, we treated OSCC cell-derived exosomes with PKH26, a fluorescent dye with long aliphatic tails that are incorporated into the lipid membrane of exosome vesicles [30]. OSC-4 cells were incubated with PKH26-labeled OSC-4-derived exosomes for 4 h. We observed the presence of PKH26-positive granules in the cytoplasm of OSC-4 cells by confocal laser microscopy, suggesting that OSCC cells secrete exosomes and uptake their own exosomes (Fig 2).

### OSCC cell-derived exosomes promote tumor cell proliferation, migration, and invasion

**Fig 3:**
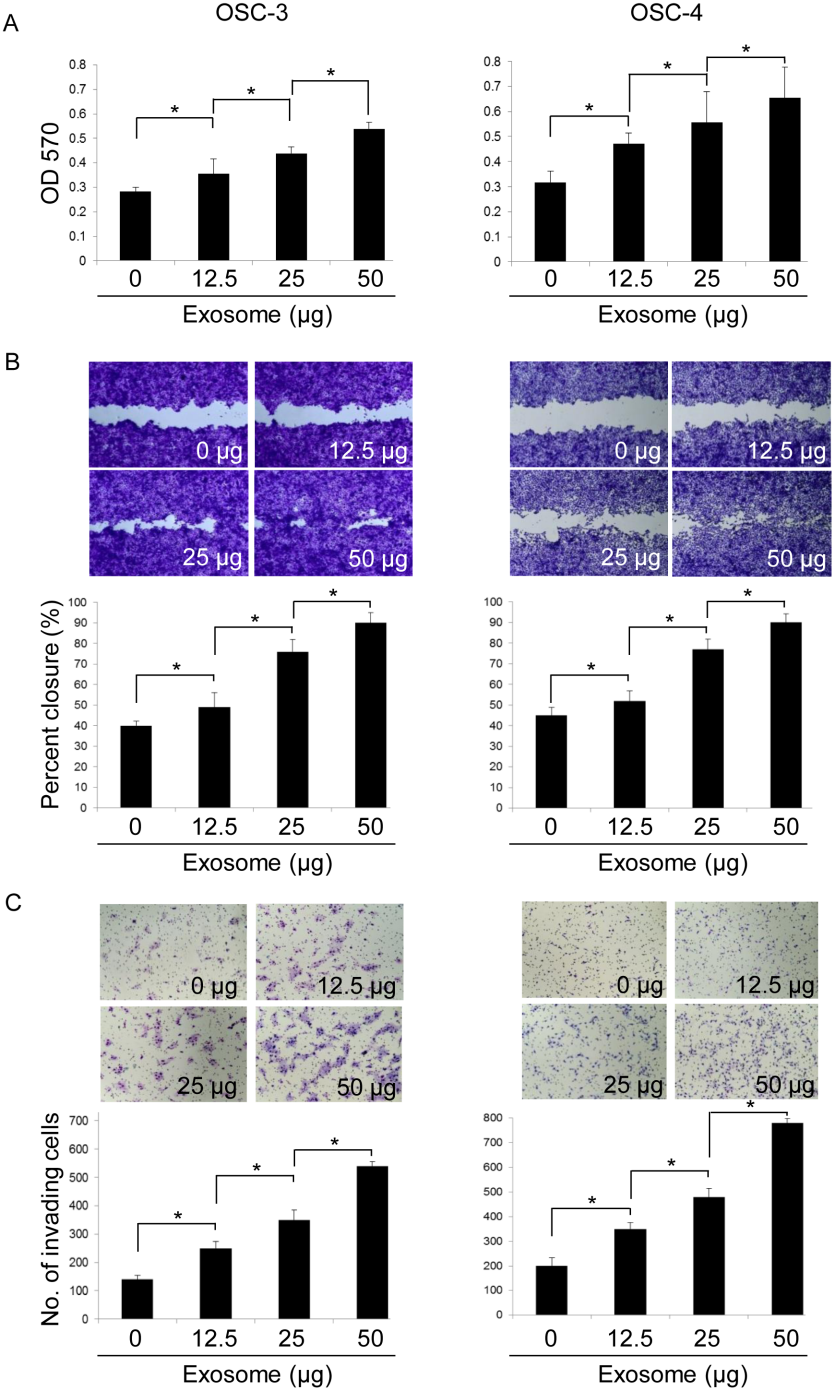
Effects of OSCC cell-derived exosomes on proliferation, migration, and invasion of OSCC cells. OSCC cell lines were incubated in the presence or absence of exosomes (12.5, 25, and 50 μg) for 24 (A), 10 (B), or 18 h (C). The viable (A), migrating (B), and invading (C) cell numbers were then determined by using the MTT, wound healing, and invasion assay, respectively. The values are presented as the mean ± SD; n = 3 for each group. * p < 0.05 against control OSCC cells, by Mann–Whitney's U-test.

To determine the autocrine or paracrine effects of OSCC cell-derived exosomes *in vitro*, we determined the effects of OSCC cell-derived exosomes on OSCC cell behavior. Exosomes derived from OSC-3 and OSC-4 cells promoted the proliferation of each OSCC cell line in a dose-dependent manner (Fig 3A and S1 Fig). Furthermore, the effect of OSCC cell-derived exosomes on cell migration was examined using a wound healing assay. Exosomes derived from OSC-3 and OSC-4 cells also promoted the migration of each OSCC cell line in a dose-dependent manner. Treatment of the cells with 50 μg exosomes induced approximately 90% closure of the wound field (Fig 3B). In addition, the effect of the exosomes on invasion was evaluated by using an invasion chamber assay. As the concentration of exosomes increased, the invasion of OSC-3 and OSC-4 cells was significantly promoted (Fig 3C). Therefore, OSCC cells may promote their own progression through the secretion and uptake of OSCC cell-derived exosomes.

### OSCC cell-derived exosomes activate the PI3K/Akt, MAPK/ERK, and JNK-1/2 pathways

**Fig 4:**
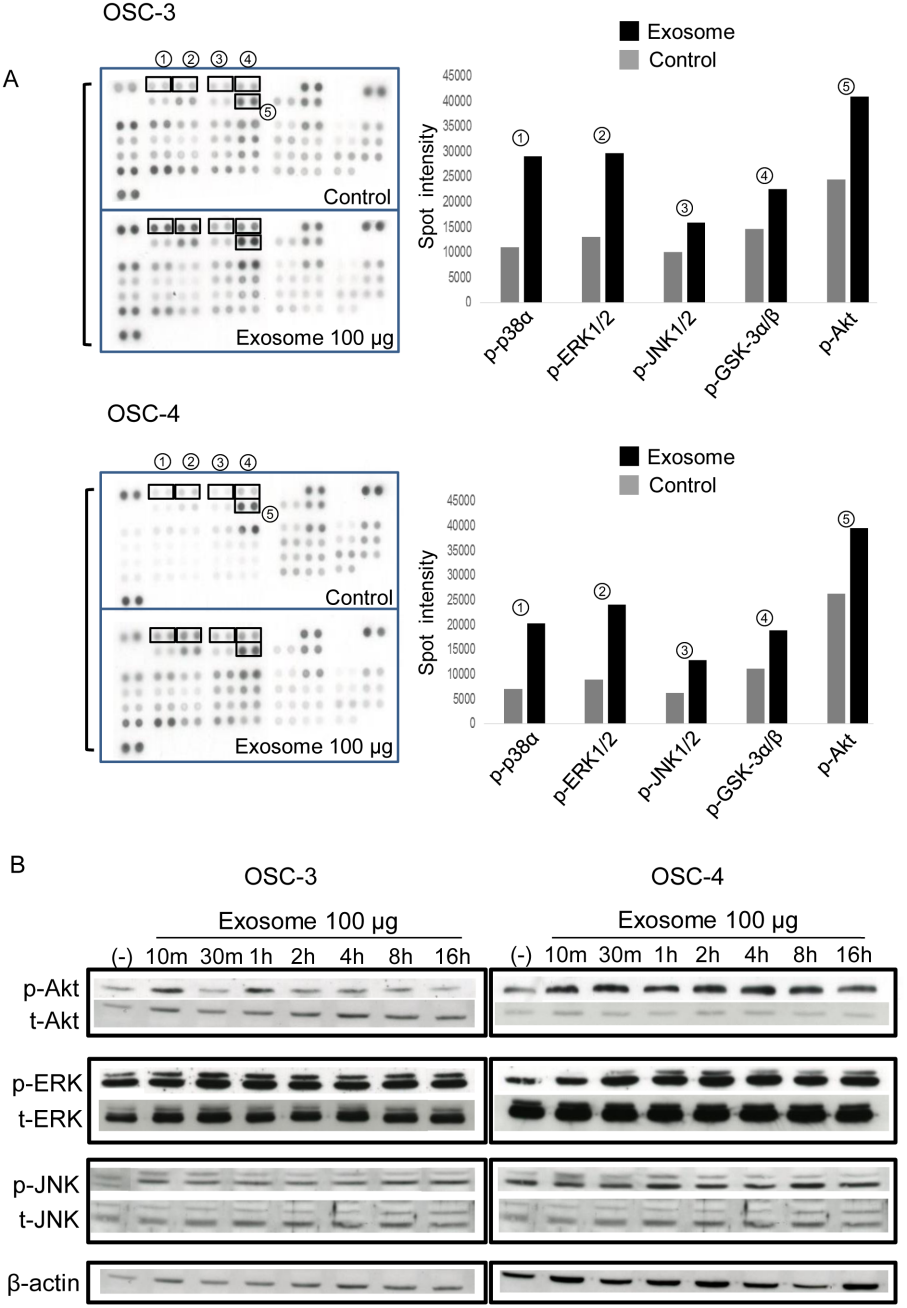
Effects of OSCC cell-derived exosomes on the activation of signal transduction proteins. (A) The phosphorylation of kinases in whole cell lysate from OSC-3 and OSC-4 cells in the presence or absence of 100 μg exosomes was analyzed by Proteome Profiler™ Human Phospho-Kinase Array Kit. The five representative molecules, which were about 50% more hyperphosphorylated in the treated cells than in the control cells are shown in the right panel. (B) Time course of the expression of p-Akt, p-ERK, and p-JNK induced by exosomes in OSCC cells. Cells were exposed to 100 μg exosomes for the indicated periods and total cell lysate was subjected to western blotting analysis.

To investigate OSCC cell-derived exosome-activated signal transduction, we compared the relative phosphorylation levels of protein kinases in exosome-treated OSCC cells. As shown in Fig 4A, an increase in the phosphorylation of p-38α, ERK1/2, JNK1/2, GSK-3α/β, and Akt was observed in OSC-3 and OSC-4 cells treated with each OSCC cell-derived exosomes for 1 h. In OSC-3 cells, exosomes rapidly induced the phosphorylation of p-38α (2.63 fold), ERK1/2 (2.26 fold), JNK1/2 (1.61 fold), GSK-3α/β (1.53 fold), and Akt (1.67 fold). In OSC-4 cells, exosomes also induced the phosphorylation of p-38α (2.85 fold), ERK1/2 (2.67 fold), JNK1/2 (2.04 fold), GSK-3α/β (1.68 fold), and Akt (1.54 fold). Next, we examined the time course of exosome-induced phosphorylation levels of these signaling proteins by western blot analysis. In both OSC-3 and OSC-4 cells, hyper-phosphorylation of ERK and JNK induced by exosomes lasted 16 h. However, the phosphorylation of Akt was obvious after 10 min and 1 h in OSC-3 cells; in OSC-4 cells, it was upregulated within 10 min and maintained for 16 h (Fig 4B).

**Fig 5:**
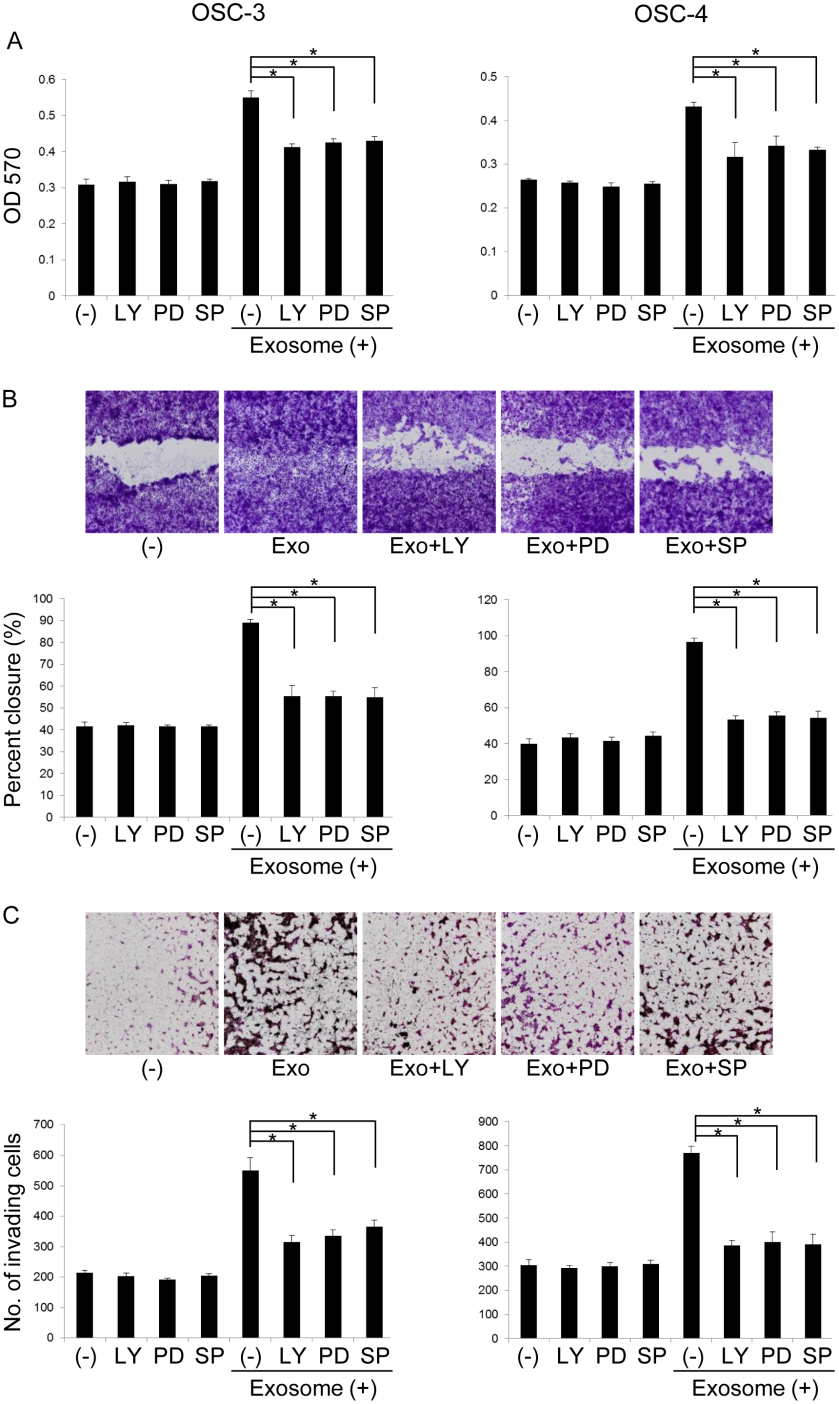
Effects of kinase inhibition on OSCC cell-derived exosome-induced proliferation, migration, and invasion of OSCC cells. OSCC cell lines were treated with 10 μM LY 294002 (LY), 50 μM PD98059 (PD), or 2.5 μM SP600125 (SP) in the presence or absence of 50 μg exosomes for 24 h (A), 10 h (B), or 18 h (C). The viable (A), migrating (B), and invading (C) cell numbers were then determined by using the MTT, wound healing, and invasion assay, respectively. The values are presented as the mean ± SD; n = 3 for each group. * p < 0.05 against control OSCC cells, by Mann–Whitney's U-test.

To ascertain the involvement of the Akt, ERK, and JNK signaling pathways in OSCC cell-derived exosome-induced promotion of proliferation, migration, and invasion, we used LY294002, PD98059, and SP600125, inhibitors of the Akt, ERK, and JNK pathways, respectively. Exosome-induced cell proliferation was partially inhibited by the treatment with these kinase inhibitors (Fig 5A and S2 Fig). These inhibitors did not affect the proliferation of control cells. In addition, the effects of OSCC cell-derived exosomes on migration and invasion were partially abolished by the addition of these inhibitors (Fig 5B and 5C). These results suggest that OSCC cell-derived exosomes promote tumor cell proliferation, migration, and invasion of OSCC cells through activation of the Akt, ERK, and JNK signaling pathways.

### OSCC cell-derived exosomes promote tumor growth *in vivo*

**Fig 6:**
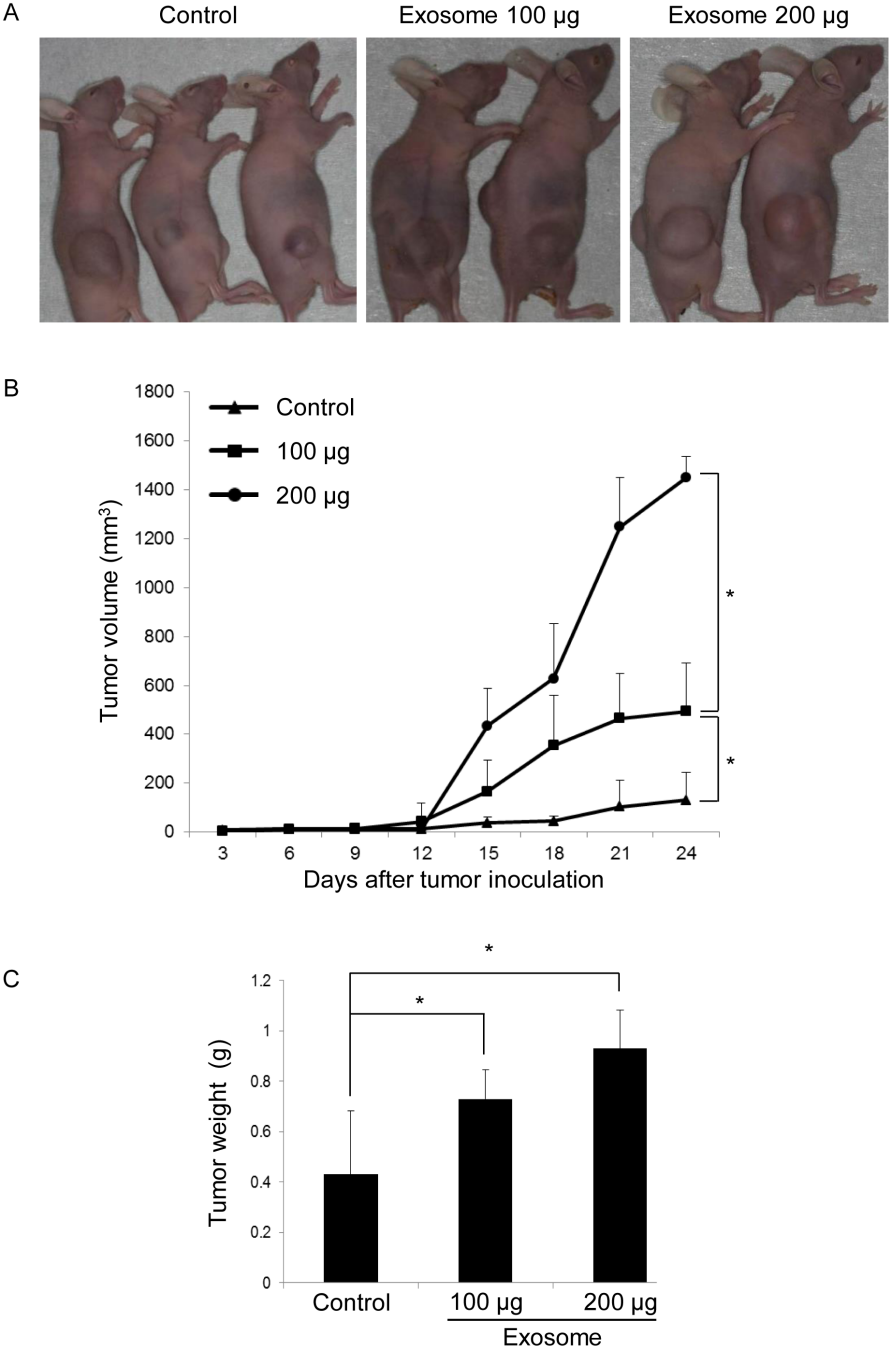
Effects of OSCC cell-derived exosomes on tumor growth in a xenograft model. BALB/c nude mice were treated as described in Materials and Methods. Tumor volume of OSC-4 cells xenografted to the back of the mice was estimated for 27 days. (A) Photographs of OSC-4 tumor-bearing mice were taken at euthanasia. (B) Tumor size was measured and calculated every 3 days. (C) Tumor weight at euthanasia for control and exosome-treated mice. The values are presented as the mean ± SD; n = 8 for each group. *p < 0.05 against control tumor by Mann-Whitney’s U-test.

To further assess the role of OSCC cell-derived exosomes in tumor growth *in vivo*, we established a tumor mouse model using 5-week-old BALB/c mice by subcutaneously injecting OSC-4 cells alone or OSC-4 cells with OSC-4 cell-derived exosomes (100 μg and 200 μg, respectively) into the animal back. In the exosome co-implantation group, tumor growth was significantly promoted in a dose-dependent manner (Fig 6A and 6B). Tumor weight was also measured after euthanasia. There were significant differences in tumor weight between the exosome co-implantation group and control group (Fig 6C), suggesting that OSCC cell-derived exosomes effectively promote tumor growth *in vivo*.

### Exosome uptake by OSCC cells can be blocked by treatment with heparin

**Fig 7:**
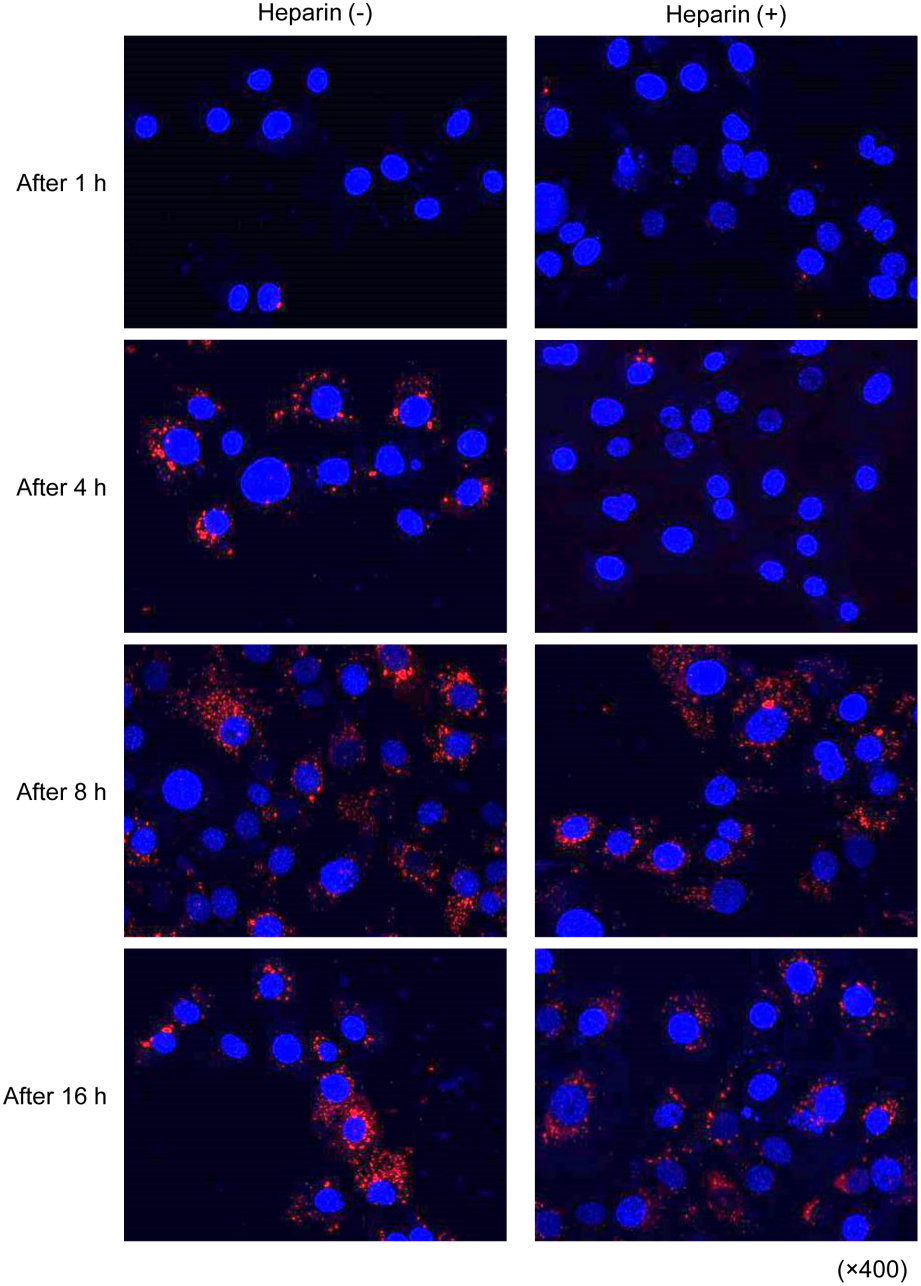
Effects of heparin on cellular internalization of OSCC cell-derived exosomes into OSCC cells. OSC-4 cells were pretreated with 10 μg/mL heparin for 1 h and then cultured in the presence of 100 μg of PKH26 (red)-labeled exosomes from OSC-4 cells for 1, 4, 8, and 16 h and analyzed by confocal microscopy. Nuclei were stained with DAPI (blue).

OSCC cells promote tumor progression through the secretion and uptake of their own exosomes. Thus, we then focused on the inhibition of the exosome uptake into tumor cells themselves as one strategy for cancer treatment, targeting tumor cell-derived exosomes. Exosomes interact with and are taken up by cells in many ways, including receptor-mediated endocytosis, phagocytosis, macropinocytosis, and direct fusion with the plasma membrane [31]. Cancer cell exosomes are internalized and taken up depending on the presence of cell-surface heparin sulfate proteoglycans (HSPGs) and heparin is a competitive inhibitor of cell surface receptors dependent on HSPG coreceptors [25, 32]. Thus, we investigated whether the exosome uptake by OSCC cells could be inhibited by heparin. We pretreated OSC-4 cells with 10 μg/mL heparin for 1 h and then cultured these cells in the presence of exosomes for each indicated time. The results show that the uptake of exosomes by OSC-4 cells was suppressed by pretreatment with heparin for up to 4 h. The suppressive effect of heparin was abolished at 8 and 16 h (Fig 7).

### Heparin inhibits OSCC cell-derived exosomes-induced tumor progression

**Fig 8:**
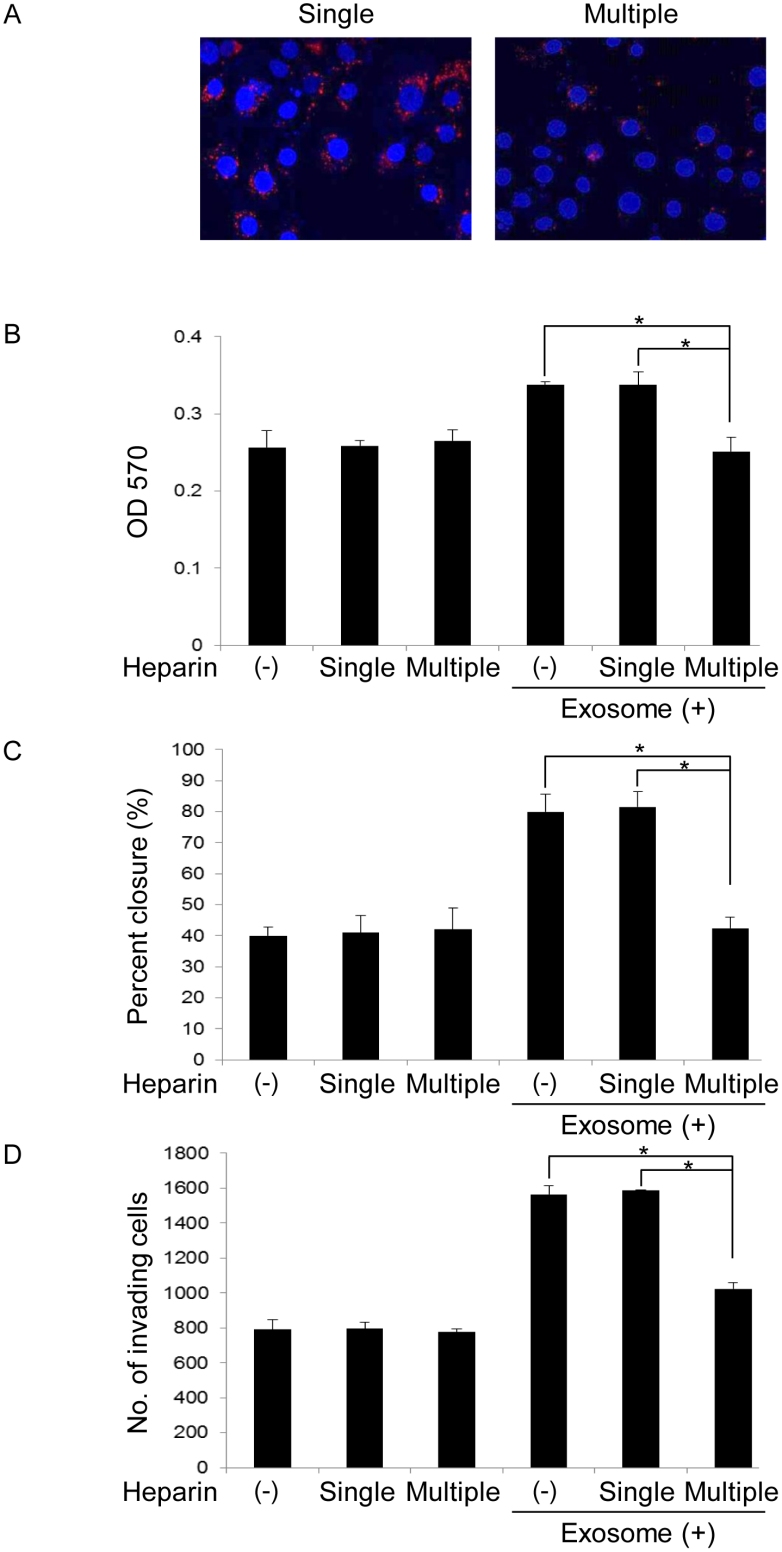
Effects of heparin on OSCC cell-derived exosome-induced proliferation, migration, and invasion of OSCC cells. OSCC cell lines were treated with 10 μg/mL heparin only once at the beginning of the experiment (single administration group) or every 4 h (multiple administration group) in the presence or absence of 50 μg exosomes for 16 h (A), 24 h (B), 10 h (C), or 18 h (D). Cellular internalization of OSCC cell-derived exosomes into OSC-4 cells was analyzed by confocal maicroscopy (A). The viable (B), migrating (C), and invading (D) cell numbers were then determined by the MTT, wound healing, and invasion assay, respectively. The values are presented as the mean ± SD; n = 3 for each group. * p < 0.05 against control of OSCC cells, by Mann–Whitney's U-test.

Heparin treatment effectively inhibited the uptake of OSCC derived-exosomes by OSCC cells, but the effect was transient. Next, we compared the effects of single and multiple heparin treatments on tumor progression. The uptake of exosomes by OSC-4 cells was not suppressed by single heparin administration. On the other hands, the suppressive effect of heparin was observed by multiple treatments (Fig 8A). Single heparin administration did not influence the proliferation, migration, and invasion of OSCC cells regardless of the presence or absence of exosomes. However, multiple heparin treatments significantly inhibited the proliferation, migration, and invasion induced by OSCC cell-derived exosomes (Fig 8B, 8C and 8D).

**Fig 9:**
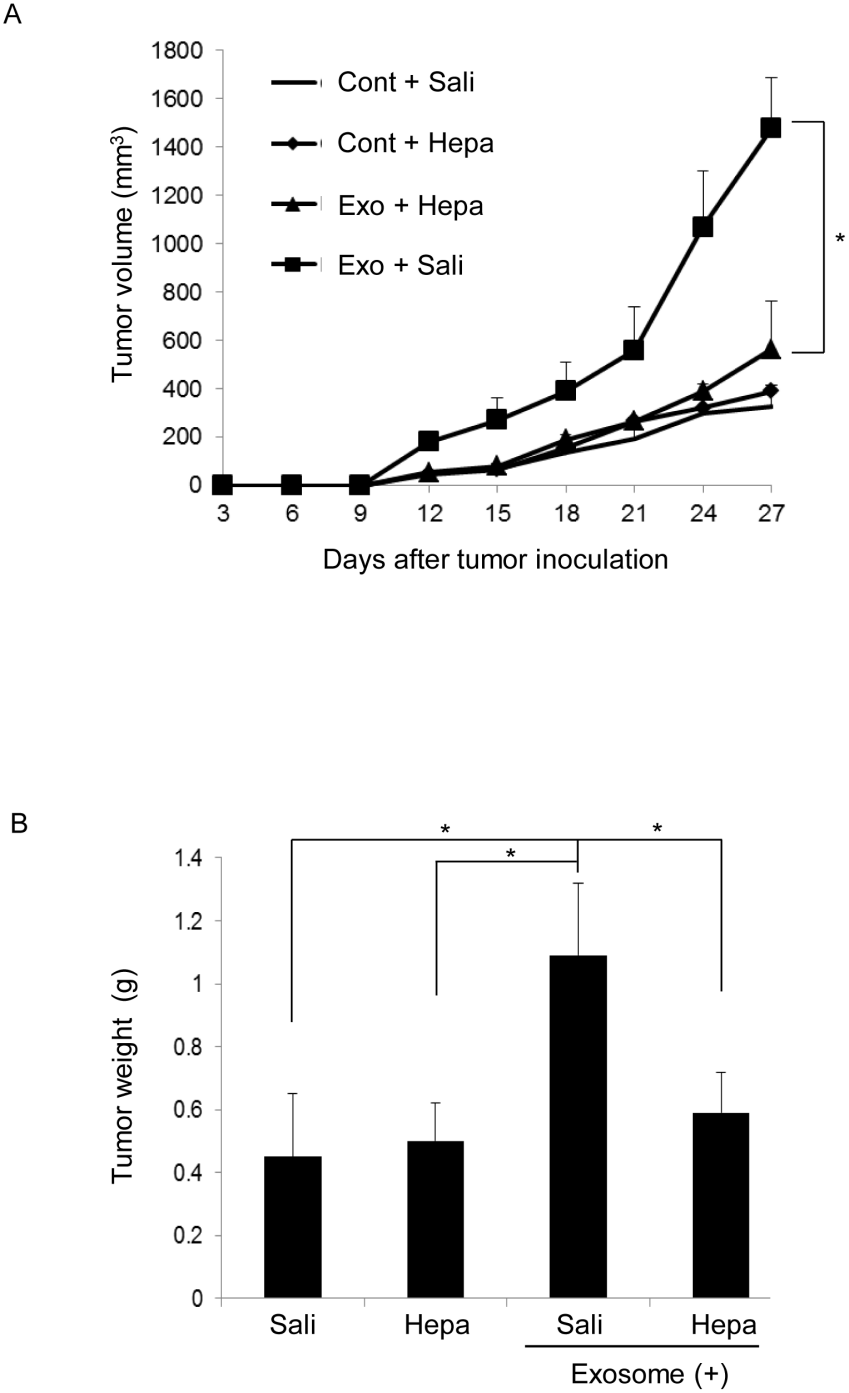
Effects of heparin on OSCC cell-derived exosome-induced tumor growth in xenograft models. BALB/c nude mice were treated as described in Materials and Methods. Tumor volume of OSC-4 cells xenografted to the back of mice was estimated for 27 days. (A) Tumor size was measured and calculated every 3 days. (B) Tumor weight at euthanasia for control and exosome-treated mice. The values are presented as the mean ± SD; n = 8 for each group. *p < 0.05 against control tumor by Mann-Whitney’s U-test. Hepa means heparin, and Sali means saline.

Finally, we examined the effects of heparin on exosome-induced tumor growth *in vivo*. The tumor growth in the exosome co-implantation group was significantly inhibited by continuous delivery of heparin by an osmotic pump (Fig 9). These results suggest that continuous administration of heparin is needed to inhibit tumor progression induced by OSCC cell-derived exosomes.

## Discussion

Cell–cell communication is crucial for the regulation of various biological phenomena in multicellular organisms, including development and homeostasis. Especially, interactions among tumor cells in the tumor microenvironment are essential for tumor cell growth, invasion, drug resistance, and metastasis [33]. Therefore, understanding these cell–cell interactions is important for the exploration of new cancer therapies. Tumor cells secrete several molecules that contribute to cancer development, including hormones, growth factors, cytokines, and chemokines. Thus, some molecular targeted therapies have been developed and applied clinically [34–36]. However, important factors involved in these complex interactions have not been completely identified [37]. It recently became clear that exosomes released from different cell types act as mediators of cell-cell communication. In the present study, we investigated effects of OSCC cell-derived exosomes on the malignant potential of tumor cells. Exosomes isolated and purified from the supernatant of OSC-3 and -4 cells showed similar morphological features to those from other sources and expressed certain exosome marker proteins such as CD9 and CD63 [38]. We demonstrated that OSC-3 and -4 cell-derived exosomes facilitated the proliferation, migration, and invasion of OSC-3 and -4 cells from which they originated in a dose-dependent manner. These results coincide with previous studies reporting that breast, gastric, and bladder cancer cell-derived exosomes induced the progression of their parental cells *in vitro* [39–41]. Thus, OSCC cell-derived exosomes may exhibit crucial tumor growth-promoting effects involved in cancer progression in an autocrine or paracrine fashion.

Next, we examined the molecular signaling activated by exosomes in OSCC cells. PI3K/Akt and MAPK/ERK signaling pathways are involved in the survival of a wide range of cell types. Gastric and bladder cancer cell-derived exosomes promoted tumor cell proliferation through the activation of PI3K/Akt and MAPK/ERK signaling pathways [39, 41]. In this study, we revealed that OSCC cell-derived exosomes also activated the phosphorylation of p-38α, ERK1/2, JNK1/2, GSK-3α/β, and Akt and that pharmacological inhibitors of PI3K (LY294002), ERK-1/2 (PD98059), and JNK-1/2 (SP600125) abolished the tumor-promoting effects induced by OSCC cell-derived exosomes. The results showed that the activation of Akt, ERK, and JNK signaling pathways was essential for the exosome-induced effects. In other words, exosomes have emerged as novel subcellular transduction materials for signal pathways, such as PI3K associated pathway, and regulated malignant transformation of recipient cells. OSCC cells may acquire more malignant characteristics through exogenous OSCC cells-derived exosomes-induced activation of PI3K/Akt signaling pathways, since this signaling pathways are associated with cellular transformation, tumorigenesis, cancer progression, and drug resistance through the regulation of oncogene and gate keeper tumor suppressor gene expression (c-Myc, p53, p27^Kip1^, and p21^cip1/WAF1^). It has been reported that PI3K/Akt signaling is hyperactivated in human cancers including OSCC [42]. During oral carcinogenesis, it is speculated that exosomes secreted by the initial small number of malignant transformed oral keratinocytes are incorporated by the cell of origin or surrounding cells and promote cancer progression through PI3K signaling regulated-acceleration of DNA damage/mutation or loss of caretaking/nuclear stability [43].

Because the functional effects of exosomes mostly rely on internalization and subsequent release of the exosome content in recipient cells, the elucidation and targeting of exosome uptake mechanisms remain an important challenge. Experimentally supported hypotheses include receptor-ligand interaction, fusion with the plasma membrane, and internalization of the exosomes by the recipient cells via endocytosis [30]. Recently, the small GTPase Rab27a and a syndecan-syntenin-ALIX-mediated pathway were reported to regulate exosomal biogenesis and secretion [21, 44, 45]. Furthermore, Christianson *et al*. demonstrated that HSPGs, a family of proteins substituted with glycosaminoglycan polysaccharides, function as receptors of cancer cell-derived exosomes, and the uptake was inhibited by heparin in human glioblastoma cells [25]. Franzen *et al*. also reported that exosome uptake by recipient cells was dependent on the dose of exosomes and treatment time, which can be partially blocked by heparin treatment in bladder cancer cells [32]. In our study, similar to these reports, heparin effectively inhibited the uptake of OSC-4 cell-derived exosomes by OSC-4 cells and their subsequent effects such as promotion of cell proliferation, migration, and invasion. Heparan sulfate binding proteins include cell surface proteins, extracellular matrix proteins, growth factors, cytokines, chemokines, enzymes, and enzyme inhibitors. There is some documentation about the expression of syndecan-1 in OSCC, seems to be associated with the differentiation status of the tumor cells [46]. Although the cargo of OSCC-derived exosomes is not certain, these exosomes may be incorporated through the interaction between heparan sulfate binding proteins on exosomal membrane molecules such as EGFR and HSPG such as syndecan-1 expressed in OSCC cell surface. Our data indicated that the HSPG dependent entry pathway is essential for the biological activity of OSCC cell-derived exosomes and the blockage by heparin can be a therapeutic approach for OSCC treatment.

Heparin is commonly used for the prevention or treatment of venous thromboembolism in cancer patients. In addition to its antithrombotic activity, cancer patients treated with heparin showed an improved survival in a number of retrospective and prospective studies [47, 48]. Heparin functions as anti-tumor agent through inhibition of the expression of proto-oncogenes such as c-fos and c-myc and inhibition of selectin and integrin functions [49]. In our model, heparin alone did not present anti-tumor effects. The blockage of exosome uptake by heparin was transient and a sustained treatment was necessary to inhibit the exosome uptake by OSC-4 cells. This phenomenon may be caused by heparanase, a heparin-degrading enzyme. In fact, heparanase mRNA and protein expression was observed in OSCC [50]. Moreover, several investigators reported that heparanase activity was high in tumor cells and involved in cancer metastasis and angiogenesis [51]. Therefore, the continuous application of heparin may be necessary to block the tumor-promoting effects induced by OSCC-derived exosomes. In addition, we speculate that heparin can inhibit the malignant transformation of OSCC cells induced by large amounts of exogenous exosomes, but under steady state conditions, other pathways, such as fusion with the plasma membrane, or macropinocytosis may be important for uptake of exosomes in OSCC cells.

In summary, the present study demonstrated that OSCC cells promote tumor progression through the secretion and uptake of their own exosomes. The uptake of exosomes by OSCC cells and subsequent tumor progression was abrogated in the presence of heparin. However, further clinical evaluation and analysis of primary OSCC and /or local and distant extension of clinical OSCC growth needs to be undertaken.

## Supporting Information

S1 Fig Effects of OSCC cell-derived exosomes on proliferation of OSCC cells. OSCC cell lines were incubated in the presence or absence of exosomes (12.5, 25, and 50 μg) for 24 h. The cell viability was then determined by using CyQUANT Direct Cell Proliferation Assay. The values are presented as the mean ± SD; n = 3 for each group. * p < 0.05 against control OSCC cells, by Mann–Whitney's U-test.
(TIF)

S2 Fig Effects of kinase inhibition on OSCC cell-derived exosome-induced proliferation of OSCC cells. OSCC cell lines were treated with 10 μM LY 294002 (LY), 50 μM PD98059 (PD), or 2.5 μM SP600125 (SP) in the presence or absence of 50 μg exosomes for 24 h. The cell viability was then determined by using CyQUANT Direct Cell Proliferation Assay. The values are presented as the mean ± SD; n = 3 for each group. * p < 0.05 against control OSCC cells, by Mann–Whitney's U-test.
(TIF)

## References

[1] Torre LA, Bray F, Siegel RL, Ferlay J, Lortet-Tieulent J, Jemal A. Global cancer statistics, 2012. CA Cancer J Clin. 2015;65(2): 87–108. 10.3322/caac.21262 25651787

[2] Raposo G, Stoorvogel W. Extracellular vesicles: exosomes, microvesicles, and friends. J. Cell Biol. 2013;200(4): 373–383. 10.1083/jcb.201211138 23420871 PMC3575529

[3] Vlassov AV, Magdaleno S, Setterquist R, Conrad R. Exosomes: Current knowledge of their composition, biological functions, and diagnostic and therapeutic potentials. Biochim Biophys Acta. 2012;1820(7): 940–948. 10.1016/j.bbagen.2012.03.017 22503788

[4] Mathivanan S, Ji H, Simpson RJ. Exosomes: extra-cellular organelles important in intercellular communication. J Proteomics. 2010;73: 1907–1920. 10.1016/j.jprot.2010.06.006 20601276

[5] Bang C, Thum T. Exosomes: new players in cell-cell communication. Int J Biochem Cell Biol. 2012;44(11): 2060–2064. 10.1016/j.biocel.2012.08.007 22903023

[6] Johnstone RM, Adam M, Hammond JR, Orr L, Turbide C. Vesicle formation during reticulocyte maturation. Association of plasma membrane activities with released vesicles (exosomes). J Biol Chem. 1987;262(19): 9412–9420. 3597417

[7] Potolicchio I, Carven GJ, Xu X, Stipp C, Riese RJ, Stern LJ, et al Proteomic analysis of microglia-derived exosomes: metabolic role of the aminopeptidase CD13 in neuropeptide catabolism. J Immunol. 2005;175(4): 2237–2243. 16081791 10.4049/jimmunol.175.4.2237

[8] Yang C, Robbins PD. The roles of tumor-derived exosomes in cancer pathogenesis. Clin Dev Immunol. 2011;2011: 842849 10.1155/2011/842849 22190973 PMC3235485

[9] Henderson MC, Azorsa DO. The genomic and proteomic content of cancer cell-derived exosomes. Front Oncology. 2012;2: 38. 10.3389/fonc.2012.00038 PMC3355967 22649786

[10] Principe S, Hui AB, Bruce J, Sinha A, Liu FF, Kislinger T. Tumor-derived exosomes and microvesicles in head and neck cancer: Implications for tumor biology and biomarker discovery. Proteomics. 2013;13(10–11): 1608–1623. 10.1002/pmic.201200533 23505015

[11] Somasundaram R, Herlyn M. Melanoma exosomes: messengers of metastasis. Nat Med. 2012;18(6): 853–854. 10.1038/nm.2775 22673991

[12] Taylor DD, Gercel-Taylor C. Micro RNA signatures of tumor derived exosomes as diagnostic biomarkers of ovarian cancer. Gynecol Oncol. 2008;110(1): 13–21. 10.1016/j.ygyno.2008.04.033 18589210

[13] Ristorcelli E, Beraud E, Verrando P, Villard C, Lafitte D, Sbarra V, et al Human tumor nanoparticles induce apoptosis of pancreatic cancer cells. FASEB J. 2008;22(9): 3358–3369. 10.1096/fj.07-102855 18511551

[14] Altevogt P, Bretz NP, Ridinger J, Utikal J, Umansky V. Novel insight into exosome-induced, tumor-associated inflammation and immunomodulation. Semin Cancer Biol. 2014;28: 51–57. 10.1016/j.semcancer.2014.04.008 24769223

[15] Whiteside TL. Immune modulation of T-cell and NK (natural killer) cell activities by TEXs (tumor-derived exosomes). Biochem Soc Trans. 2013;41(1): 245–251. 10.1042/BST20120265 23356291 PMC3721347

[16] Saleem SN, Abdel-Mageed. Tumor-derived exosomes in oncogenic reprogramming and cancer progression. Cell Mol Life Sci. 2015;72(1): 1–10. 10.1007/s00018-014-1710-4 25156068 PMC4282952

[17] Mrizak D, Martin N, Barjon C, Jimenez-Pailhes AS, Mustapha R, Niki T, et al Effect of nasopharyngeal carcinoma-derived exosomes on human regulatory T cells. J Natl Cancer Inst. 2014;107(1): 363 10.1093/jnci/dju363 25505237

[18] Ye SB, Li ZL, Luo DH, Huang BJ, Chen YS, Zhang XS, et al Tumor-derived exosomes promote tumor progression and T-cell dysfunction through the regulation of enriched exosomal microRNAs in human nasopharyngeal carcinoma. Oncotarget. 2014;5(14): 5439–5452. 24978137 10.18632/oncotarget.2118 PMC4170615

[19] Zhao L, Liu W, Xiao J, Cao B. The role of exosomes and “exosomal shuttle microRNA” in tumorigenesis and drug resistance. Cancer Lett. 2015;356(2 Pt B): 339–346. 10.1016/j.canlet.2014.10.027 25449429

[20] Kahlert C, Kalluri R. Exosomes in tumor microenvironment influence cancer progression and metastasis. J Mol Med. 2013;91(4): 431–437. 10.1007/s00109-013-1020-6 23519402 PMC4073669

[21] Bobrie A, Krumeich S, Reyal F, Recchi C, Moita LF, Seabra MC, et al Rab27a supports exosome-dependent and -independent mechanisms that modify the tumor microenvironment and can promote tumor progression. Cancer Res. 2012;72(19): 4920–4930. 10.1158/0008-5472.CAN-12-0925 22865453

[22] Peinado H, Aleckovic M, Lavotshkin S, Matei I, Costa-Silva B, Moreno-Bueno G, et al Melanoma exosomes educate bone marrow progenitor cells toward a pro-metastatic phenotype through MET. Nat Med. 2012;18(6): 833–891. 10.1038/nm.2753 PMC3645291 22635005

[23] Verderio C, Muzio L, Turola E, Bergami A, Novellino L, Ruffini F, et al Myeloid microvesicles are a marker and therapeutic target for neuroinflammation. Ann Neurol. 2012;72(4): 610–624. 10.1002/ana.23627 23109155

[24] Zhang Y, Roccaro AM, Rombaoa C, Flores L, Obad S, Fernandes SM, et al LNA-mediated anti-miR-155 silencing in low-grade B-cell lymphomas. Blood. 2012;120(8): 1678–1686. 10.1182/blood-2012-02-410647 22797699

[25] Christianson HC, Svensson KJ, van Kuppevelt TH, Li JP, Belting M. Cancer cell exosomes depend on cell-surface heparan sulfate proteoglycans for their internalization and functional activity. Proc Natl Acad Sci USA. 2013;110(43): 17380–17385. 10.1073/pnas.1304266110 24101524 PMC3808637

[26] Ueta E, Umazume M, Yamamoto T, Osaki T. Tumorigenicity of cell lines established from oral squamous cell carcinoma and its metastatic lymph nodes. Eur J Cancer B Oral Oncol. 1994;30B(5): 296–301. 7703799 10.1016/0964-1955(94)90028-0

[27] Lin R, Wang S, Zhao RC. Exosomes from human adipose-derived mesenchymal stem cells promote migration through Wnt signaling pathway in a breast cancer cell model. Mol Cell Biochem. 2013;383(1–2): 13–20. 10.1007/s11010-013-1746-z 23812844

[28] Jiang H, Fan D, Zhou G, Li X, Deng H. Phosphatidylinositol 3-kinase inhibitor (LY294002) induces apoptosis of human nasopharyngeal carcinoma in vitro and in vivo. J Exp Clin Cancer Res. 2010;29: 34 10.1186/1756-9966-29-34 20412566 PMC2873422

[29] Lee JK, Park SR, Jung BK, Jeon YK, Lee YS, Kim MK, et al Exosomes derived from mesenchymal stem cells suppress angiogenesis by down-regulating VEGF expression in breast cancer cells. PLoS One. 2013;8(12): e84256 10.1371/journal.pone.0084256 24391924 PMC3877259

[30] Kawamoto T, Ohga N, Akiyama K, Hirata N, Kitahara S, Maishi N, et al Tumor-derived microvesicles induce proangiogenic phenotype in endothelial cells via endocytosis. PLoS One. 2012;7(3): e34045 10.1371/journal.pone.0034045 22479517 PMC3316594

[31] Escrevente C, Keller S, Altevogt P, Costa J. Interaction and uptake of exosomes by ovarian cancer cells. BMC Cancer 2011;11: 108 10.1186/1471-2407-11-108 21439085 PMC3072949

[32] Franzen CA, Simms PE, Van Huis AF, Foreman KE, Kuo PC, Gupta GN. Characterization of uptake and internalization of exosomes by bladder cancer cells. Biomed Res Int. 2014;2014: 619829 10.1155/2014/619829 24575409 PMC3915764

[33] Sun Y, Liu J. Potential of cancer cell-derived exosomes in clinical application: a review of recent research advances. Clin Ther. 2014;36(6): 863–872. 10.1016/j.clinthera.2014.04.018 24863262

[34] Melero-Martin JM, Dudley AC. Concise review: vascular stem cells and tumor angiogenesis. Stem Cells. 2011;29(2): 163–168. 10.1002/stem.583 21732475 PMC3083523

[35] Hall K, Ran S. Regulation of tumor angiogenesis by the local environment. Front Biosci. 2010;15: 195–212. 10.2741/3615 20036815

[36] Stevenson CE, Nagahashi M, Ramachandran S, Yamada A, Bear HD, Takabe K. Bevacizumab and breast cancer: what does the future hold? Future Oncol. 2012;8(4): 403–414. 10.2217/fon.12.22 22515444 PMC3464486

[37] Kosaka N, Yoshioka Y, Tominaga N, Hagiwara K, Katsuda T, Ochiya T. Dark side of the exosome: the role of the exosome in cancer metastasis and targeting the exosome as a strategy for cancer therapy. Future Oncol. 2014;10(4): 671–681. 10.2217/fon.13.222 24754596

[38] Mulcahy LA, Pink RC, Carter DR. Routes and mechanisms of extracellular vesicle uptake. J Extracell Vesicles. 2014;3 10.3402/jev.v3.24641 PMC4122821 25143819

[39] Qu JL, Qu XJ, Zhao MF, Teng YE, Zhang Y, Hou KZ, et al Gastric cancer exosomes promote tumor cell proliferation through PI3K/Akt and MAPK/ERK activation. Dig Liver Dis. 2009;41(12): 875–880. 10.1016/j.dld.2009.04.006 19473897

[40] Koga K, Matsumoto K, Akiyoshi T, Kubo M, Yamanaka N, Tasaki A, et al Purification, characterization and biological significance of tumor-derived exosomes. Anticancer Res. 2005;25(6A): 3703–3707. 16302729

[41] Yang L, Wu XH, Wang D, Luo CL, Chen LX. Bladder cancer cell-derived exosomes inhibit tumor cell apoptosis and induce cell proliferation in vitro. Mol Med Rep. 2013;8(4): 1272–1278. 10.3892/mmr.2013.1634 23969721

[42] Vander Broek R, Mohan S, Eytan DF, Chen Z, Van Waes C. The PI3K/Akt/mTOR axis in head and neck cancer: functions, aberrations, cross-talk, and therapies. Oral Dis. 2015;21(7):815–825. 10.1111/odi.12206 24219320

[43] Segrelles C, Moral M, Lara MF, Ruiz S, Santos M, Leis H, García-Escudero R, Martínez-Cruz AB, Martínez-Palacio J, Hernández P, Ballestín C, Paramio JM. Molecular determinants of Akt-induced keratinocyte transformation. Oncogene. 2006;25(8):1174–85. 16247457 10.1038/sj.onc.1209155

[44] Ostrowski M, Carmo NB, Krumeich S, Fanget I, Raposo G, Savina A, et al Rab27a and Rab27b control different steps of the exosome secretion pathway. Nat Cell Biol. 2010;12(1): 19–30. 10.1038/ncb2000 19966785

[45] Baietti MF, Zhang Z, Mortier E, Melchior A, Degeest G, Geeraerts A, et al Syndecan-syntenin-ALIX regulates the biogenesis of exosomes. Nat Cell Biol. 2012;14(7): 677–685. 10.1038/ncb2502 22660413

[46] Jackson LL, Wade Z, Hessler RB, Abdelsayed R, Rogers JB, Gourin CG. Quantitative analysis of syndecan-1 expression in dysplasia and squamous cell carcinoma of the oral cavity. Laryngoscope. 2007;117(5):868–871. 17473685 10.1097/MLG.0b013e318033c810

[47] Kuderer NM, Khorana AA, Lyman GH, Francis CW. A meta-analysis and systematic review of the efficacy and safety of anticoagulants as cancer treatment: impact on survival and bleeding complications. Cancer. 2007;110(5): 1149–1161. 17634948 10.1002/cncr.22892

[48] Tagalakis V, Blostein M, Robinson-Cohen C, Kahn SR. The effect of anticoagulants on cancer risk and survival: systematic review. Cancer Treat Rev. 2007;33(4): 358–368. 17408861 10.1016/j.ctrv.2007.02.004

[49] Bendas G, Borsig L. Cancer cell adhesion and metastasis: selectins, integrins, and the inhibitory potential of heparins. Int J Cell Biol. 2012;2012: 676731 10.1155/2012/676731 22505933 PMC3296185

[50] Vlodavsky I, Friedmann Y, Elkin M, Aingorn H, Atzmon R, Ishai-Michaeli R, et al Mammalian heparanase: gene cloning, expression and function in tumor progression and metastasis. Nat Med. 1999;5: 793–802. 10395325 10.1038/10518

[51] Vlodavsky I, Friedmann Y. Molecular properties and involvement of heparanase in cancer metastasis and angiogenesis. J Clin Invest. 2001;108: 341–347. 11489924 10.1172/JCI13662 PMC209369

