# Supplementary figures and images for: Application of a Persistent Heparin Treatment Inhibits the Malignant Potential of Oral Squamous Carcinoma Cells Induced by Tumor Cell-Derived Exosomes
Source: PLoS One. 2016 Feb 5;11(2):e0148454. doi: 10.1371/journal.pone.0148454 (PMC4743844; doi:10.1371/journal.pone.0148454)

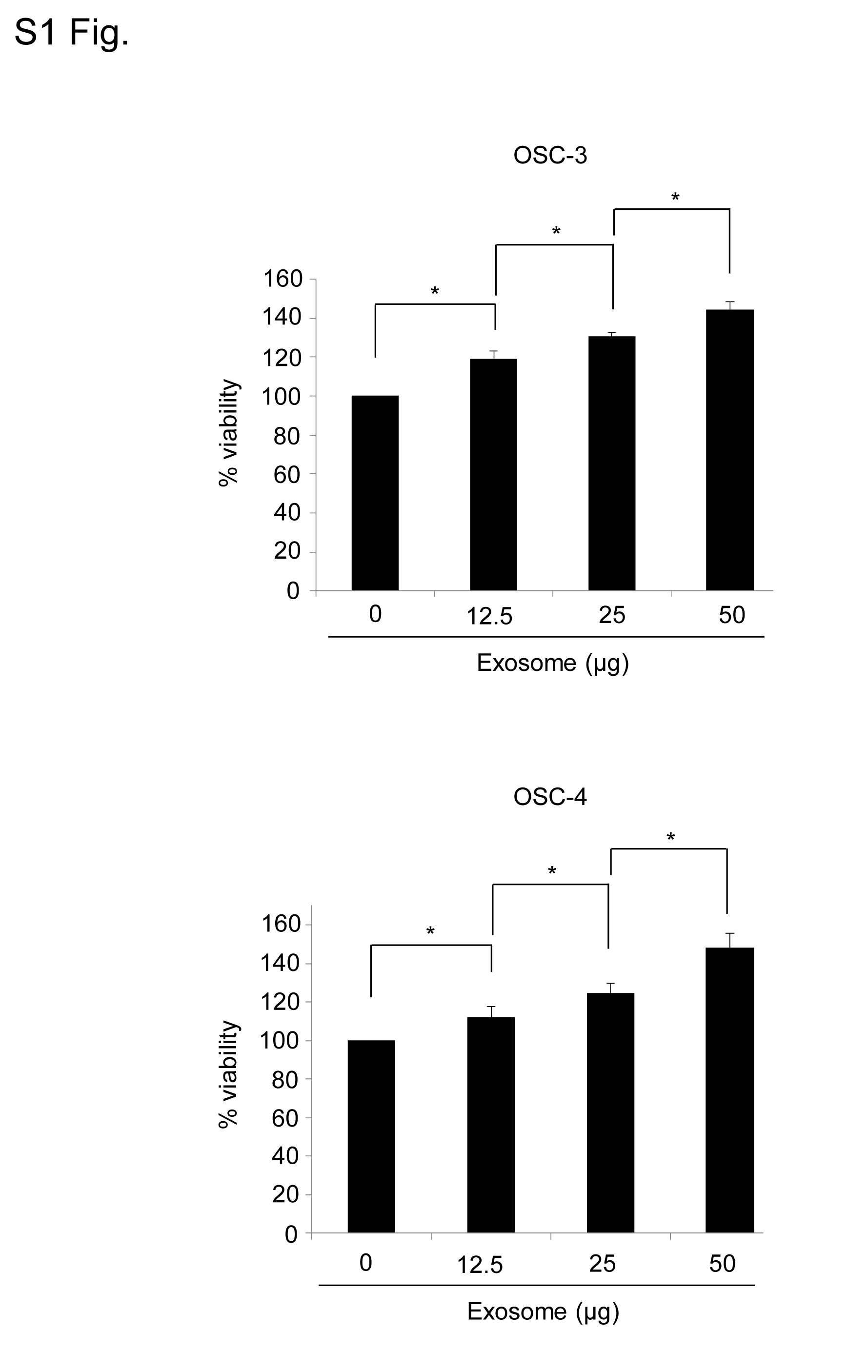

Supplement: S1 Fig — OSCC cell lines were incubated in the presence or absence of exosomes (12.5, 25, and 50 μg) for 24 h. The cell viability was then determined by using CyQUANT Direct Cell Proliferation Assay. The values are presented as the mean ± SD; n = 3 for each group. * p < 0.05 against control OSCC cells, by Mann–Whitney's U-test. (TIF) [file pone.0148454.s001.tif]

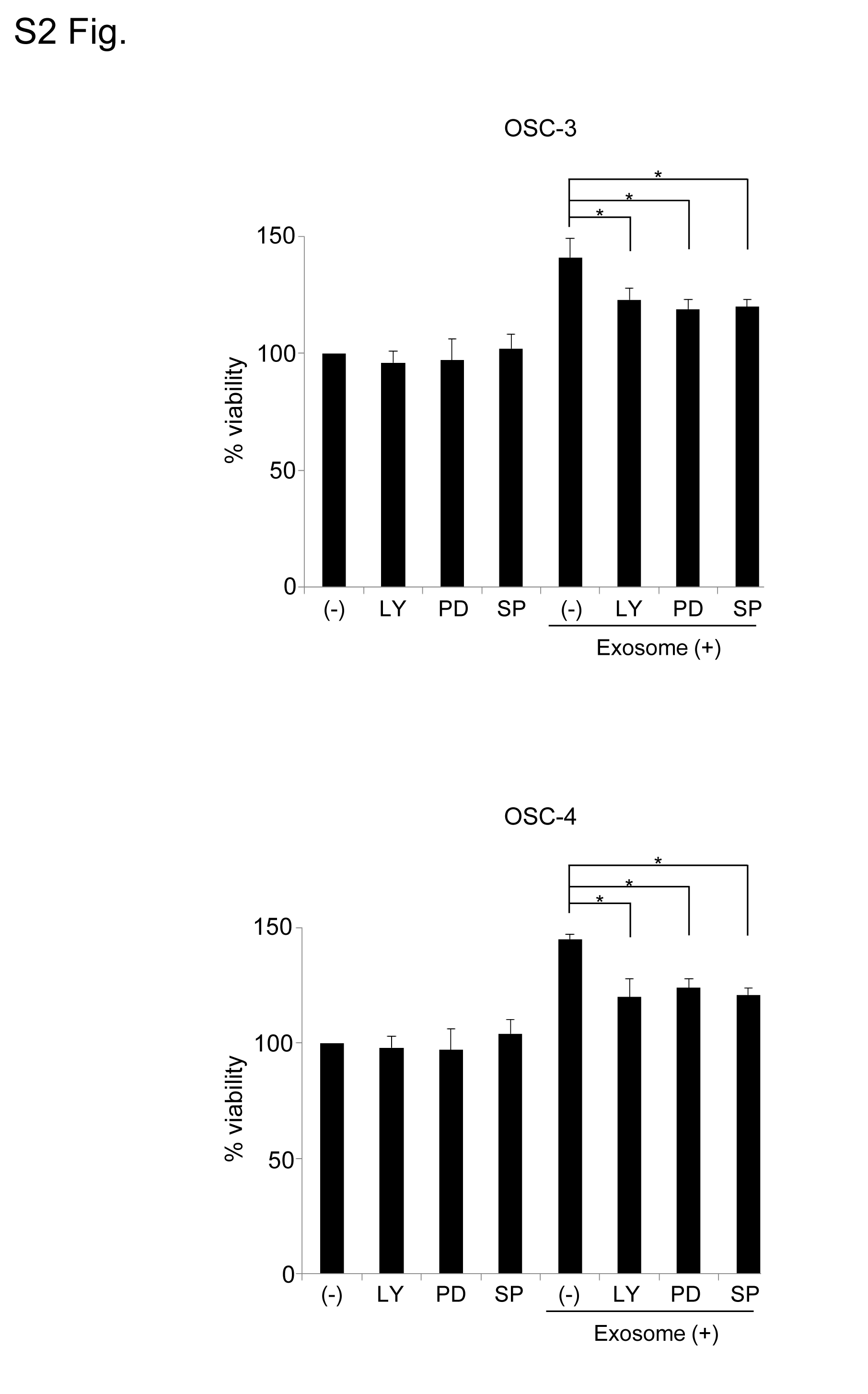

Supplement: S2 Fig — OSCC cell lines were treated with 10 μM LY 294002 (LY), 50 μM PD98059 (PD), or 2.5 μM SP600125 (SP) in the presence or absence of 50 μg exosomes for 24 h. The cell viability was then determined by using CyQUANT Direct Cell Proliferation Assay. The values are presented as the mean ± SD; n = 3 for each group. * p < 0.05 against control OSCC cells, by Mann–Whitney's U-test. (TIF) [file pone.0148454.s002.tif]
